# Evaluation of IR Biotyper for *Lactiplantibacillus plantarum* Typing and Its Application Potential in Probiotic Preliminary Screening

**DOI:** 10.3389/fmicb.2022.823120

**Published:** 2022-03-24

**Authors:** Xiaoqiong Li, Liying Zhu, Xin Wang, Jinjun Li, Biao Tang

**Affiliations:** ^1^State Key Laboratory for Managing Biotic and Chemical Threats to the Quality and Safety of Agro-Products, Institute of Food Sciences, Zhejiang Academy of Agricultural Sciences, Hangzhou, China; ^2^Institute of Agro-Product Safety and Nutrition, Zhejiang Academy of Agricultural Sciences, Hangzhou, China

**Keywords:** IR Biotyper, *Lactiplantibacillus plantarum*, probiotic screening, genotyping, phenotypic, strain typing

## Abstract

IR Biotyper (IRBT), which is a spectroscopic system for microorganism typing based on Fourier transform infrared (FTIR) technology, has been used to detect the spread of clones in clinical microbiology laboratories. However, the use of IRBT to detect probiotics has rarely been reported. Herein, we evaluated the discriminatory power of IRBT to type *Lactiplantibacillus plantarum* isolates at the strain level and explored its application potential in probiotic preliminary selection. Twenty *Lactiplantibacillus* isolates collected from pickled radishes during successive fermentation were used to test the robustness of IRBT at the strain level. IRBT was then compared with genotyping methods such as whole-genome sequencing (WGS), pulsed-field gel electrophoresis (PFGE), and multilocus sequence typing (MLST) to evaluate its discrimination power. IRBT distributed the 20 isolates into five clusters, with *L. argentoratensis* isolate C7-83 being the most distant from the other isolates, which belonged to *L. plantarum*. IRBT showed good reproducibility, although deviation in the discriminative power of IRBT was found at the strain level across laboratories, probably due to technical variance. All examined methods allowed bacterial identification at the strain level, but IRBT had higher discriminatory power than MLST and was comparable to the WGS and PFGE. In the phenotypic comparison study, we observed that the clustering results of probiotic physiological attributes (e.g., sensitivity to acid and bile salts, hydrophobicity of the cell surface, and resistance to antibiotics) were consistent with the typing results of IRBT. Our results indicated that IRBT is a robust tool for *L. plantarum* strain typing that could improve the efficiency of probiotic identification and preliminary screening, and can potentially be applied in probiotic traceability and quality control.

## Introduction

*Lactiplantibacillus plantarum* (formerly *Lactobacillus plantarum*), which is a homofermentative lactic acid bacterium (LAB), has been widely applied as a model species for ecological, metabolic, and genetic studies in lactobacilli (Zheng et al., 2020). In addition, *L. plantarum* is used as a starter culture in multiple food and beverage fermentations and as a probiotic for both animals and humans, making it of considerable academic and economic importance (das Neves Selis et al., 2021). *L. plantarum* has been classified as a “nomadic” or “generalist” bacterium because of its diverse habitats (Martino et al., 2016; Choi et al., 2018; Yu et al., 2020, 2021). Consistent with its broad environmental and host ranges, *L. plantarum* strains have significant intraspecific genetic and phenotypic versatility due to strain-specific genes (Molenaar et al., 2005; Zheng et al., 2015; Duar et al., 2017; Salvetti et al., 2018; Cen et al., 2020; Pan et al., 2021). The latest taxonomic research has elevated *L. plantarum* subsp. *plantarum* and *L. plantarum* subsp. *argentoratensis* to the species level as *L. plantarum* and *L. argentoratensis*, respectively (Liu and Gu, 2020; Zheng et al., 2020).

Food and Agriculture Organization [FAO]/World Health Organization [WHO] (2002) published the “Guidelines for Evaluation of Probiotics in Food,” the selection criteria of which included host-related stress resistance, epithelial adhesion, antimicrobial activity, and safety assessment. These aspects serve to ensure that candidate probiotics can withstand unfavorable gastrointestinal conditions, colonize intestinal epithelial cells, and contribute to host health, leading to challenging probiotic screening processes based on stringent selection criteria. In most cases, the large number of probiotic candidates lead to the use of a “step-by-step approach” involving a series of *in vitro* required tests to progressively reduce the number of candidate probiotics (de Melo Pereira et al., 2018; Yoha et al., 2021). Because the properties and benefits of *L. plantarum* are strain-specific, an easy-to-use method for fast and effective *L. plantarum* strain typing is highly desirable in both academia and industry (Fuhren et al., 2020).

Whole genome sequencing (WGS), which provides more consistent genetic information is replacing pulsed-field gel electrophoresis (PFGE) as the new “gold standard” for identifying and classifying microorganism (Neoh et al., 2019). However, regardless of their discriminatory power, the high cost, laboriousness, and time consumption of the genotypic technologies prevent their routine implementation on a large-scale basis (Quintelas et al., 2018). In addition, phenotype prediction based on genetic data is not always straightforward, and the genetic single nucleotide polymorphism (SNP) thresholds for strain delineation in bacteriology have not been universally set (Van Rossum et al., 2020). In this context, spectroscopy-based techniques such as matrix-assisted laser desorption/ionization–time of flight mass spectrometry (MALDI-TOF MS), Raman spectroscopy, and Fourier transform infrared (FTIR) have emerged as more cost-effective, convenient, and faster alternatives in bacterial strain typing (Quintelas et al., 2018).

FTIR is based on using molecular vibration fingerprints to characterize microorganisms according to strain-specific absorbance patterns in the infrared spectrum (Dinkelacker et al., 2018). The main advantages of FTIR are that it is quick, inexpensive, laboratorial simplicity, and nondestructive, has high throughput, and provides relevant information about the biomolecular contents of microorganisms, including lipids, carbohydrates, proteins, and nucleic acids, derived from IR spectra (Quintelas et al., 2018). In particular, IR Biotyper (IRBT) (Bruker Daltonik GmbH, Bremen, Germany) based on FTIR technology was launched in 2017 and emerged as a very promising system in the field of microbial strain typing, with multiple reported successful applications (de las Rivas et al., 2006; Burckhardt et al., 2019; Martak et al., 2019; Hu et al., 2020; Deidda et al., 2021). The application of FTIR is not limited to clinical and epidemiological researches but also extends to the probiotic industry (Deidda et al., 2021). Discriminating probiotics at the strain level is challenging due to their high intraspecific variation, diverse modes of action, and required manufacturing and quality control processes. Therefore, strain-specific verification of a probiotic is fundamental to quarantine its stability, quality, safety, and efficacy. Whether FTIR technology can distinguish *L. plantarum* at the strain level needs to be further confirmed.

In this study, we aim to (1) test the strain-level typing capability of IRBT for members of the *Lactiplantibacillus* genus, (2) compare the discriminatory power and concordance of IRBT with those of other genotyping techniques (WGS, PFGE, and MLST), and (3) compare the fingerprint spectra generated by IRBT with the probiotic host-associated stress resistance and safety patterns to pave the way for the introduction of this new complementary phenotypic technique into routine probiotic screening processes.

## Materials and Methods

### Isolation and Identification of *Lactiplantibacillus* Strains

The sources of the isolates are listed in Table 1. Pickled radish brines from fermentation days 7 and 21 were collected in sterile bottles and transported to the laboratory for analyses. The suspensions were serially diluted with sterile 0.9% NaCl, pour-plated on de Man, Rogosa and Sharpe (MRS) agar (Oxoid, Basingstoke, United Kingdom) and incubated at 37°C under aerobic or anaerobic conditions for 48 ± 3 h. Single-colony isolates were selected randomly and repeatedly streaked for isolation on MRS prior to characterization. All isolate identities were confirmed at the species level by MALDI-TOF MS (MALDI Biotyper; Bruker Daltonik GmbH, Bremen, Germany). Since MALDI-TOF MS scores ≤2.0 may indicate false species identification, any isolates with scores below 2.0 were additionally confirmed by 16S rRNA gene sequencing analysis. Twenty isolates of *L. plantarum* were finally selected in this study. Until the WGS comparison, it was found that isolate C7-83 belonged to *L. argentoratensis*. Isolate R106 was deposited to the China General Microbiological Culture Collection Center (CGMCC) under the deposit number CGMCC 20370. Stock cultures of each isolate were frozen at −80°C in 20% (w/v) glycerol for further studies. For phenotypic and genotypic analysis, the *Lactiplantibacillus* isolates were routinely grown in MRS medium at 37°C without shaking.

**TABLE 1:** *Lactiplantibacillus* isolates used in this study.

Isolates	Isolation information^1^	GenBank accession No.	Techniques used
C7-7	Fermented radishes; day 7	JAJCVD000000000	IRBT, PFGE, WGS, MLST, and PT
C7-35	Fermented radishes; day 7	JAJCVH000000000	IRBT, PFGE, WGS, and MLST
C7-39	Fermented radishes; day 7	JAJCVG000000000	IRBT, PFGE, WGS, MLST, and PT
C7-40	Fermented radishes; day 7	JAJCVF000000000	IRBT, PFGE, WGS, and MLST
C7-52	Fermented radishes; day 7	JAJCVE000000000	IRBT, PFGE, WGS, MLST, and PT
C7-83	Fermented radishes; day 7	JAJCVC000000000	IRBT, PFGE, WGS, MLST, and PT
R35	Fermented radishes; day 21	JAJCUY000000000	IRBT, PFGE, WGS, and MLST
R39	Fermented radishes; day 21	JAJCUX000000000	IRBT, PFGE, WGS, and MLST
R46	Fermented radishes; day 21	JAJCUW000000000	IRBT, PFGE, WGS, and MLST
R47	Fermented radishes; day 21	JAJCUV000000000	IRBT, PFGE, WGS, MLST, and PT
R49	Fermented radishes; day 21	JAJCUU000000000	IRBT, PFGE, WGS, and MLST
R58	Fermented radishes; day 21	JAJCUT000000000	IRBT, PFGE, WGS, and MLST
R62	Fermented radishes; day 21	JAJCUS000000000	IRBT, PFGE, WGS, MLST, and PT
R75	Fermented radishes; day 21	JAJCUR000000000	IRBT, PFGE, WGS, MLST, and PT
R77	Fermented radishes; day 21	JAJCUQ000000000	IRBT, PFGE, WGS, and MLST
R95	Fermented radishes; day 21	JAJCUP000000000	IRBT, PFGE, WGS, MLST, and PT
R98	Fermented radishes; day 21	JAJCUO000000000	IRBT, PFGE, WGS, MLST, and PT
R102	Fermented radishes; day 21	JAJCVB000000000	IRBT, PFGE, WGS, and MLST
R105	Fermented radishes; day 21	JAJCVA000000000	IRBT, PFGE, WGS, and MLST
R106	Fermented radishes; day 21	JAJCUZ000000000	IRBT, PFGE, WGS, MLST, and PT

*^1^On the 7th fermentation day, bacteria were isolated from spontaneous fermented radishes in three different containers, whereas on the 21st fermentation day, bacteria were isolated from fermented radishes in six different containers with or without wheat bran supplementation. IRBT, IR Biotyper; PFGE, pulsed-field gel electrophoresis; WGS, whole-genome sequencing; MLST, multilocus sequence typing; PT, phenotypic typing.*

### IR Biotyper Analysis

All isolates were grown at 37°C in MRS medium sealed with Parafilm M for 48 ± 3 h. For IRBT analysis, loading samples was prepared according to Hu et al. (2020) by the modified H_2_O-EtOH method. First, a loopful of bacterial culture was collected and resuspended in 100 μL sterile H_2_O. After vortexing, 100 μL of 70% (vol/vol) ethanol was added, and the solution was mixed by pipetting to obtain a homogeneous suspension. Then, 15 μL of bacterial suspension was spotted onto the IRBT silicon sample plate and dried at 37°C until a dry film was formed. Three or four replicates were prepared for each sample. For each run, quality control was performed with the Infrared Test Standards (IRTS 1 and 2) in the IR Biotyper kit.

Spectra were recorded in transmission mode in the spectral range of 4,000–500 cm^–1^ (mid-IR) using an IR Biotyper spectrometer (Bruker Optics-Daltonics GmbH). The spectra were then acquired, visualized, and processed by OPUS v7.5 software (Bruker Optics GmbH). Then, second-derivative FTIR spectra in the polysaccharide absorption region (1,200–9,00 cm^–1^), which is the default setting of the manufacturer, were vector-normalized and used to amplify differences between isolates and correct variations related to spectral acquisition. Data that did not meet the default quality criteria were excluded from further analyses. Hierarchical clustering analysis (HCA) of the second-derivative IRBT to illustrate the relationships between individual strains was performed offline using IRBT Client Software v2.0 (Bruker Daltonik GmbH). Dendrograms were constructed using Euclidian distances and the average linkage clustering method. For each dataset explored, the IR Biotyper software automatically calculated a clustering cut-off value, which was the result of Simpson’s index of diversity and the mean coherence of the parameter defined by the user.

Reproducibility analysis of IRBT was performed since FTIR spectroscopy techniques are known to be sensitive to variations in culture media, incubation time, temperature, and hygrometry. We evaluated the reproducibility by analyzing the 20 isolates in two laboratories (the State Key Laboratory for Managing Biotic and Chemical Threats to the Quality and Safety of Agro-Products, and the Clinical Microbiology Laboratory, School of Medicine, The Second Affiliated Hospital of Zhejiang University) independently three times on separate days.

### Genotypic Analysis

Genotyping methods such as WGS, PFGE, and MLST were incorporated to compare their discrimination power and accuracy with IRBT (PFGE and MLST subsections refer to Supplementary Material).

Genomic DNA was extracted from the *Lactiplantibacillus* isolates using a bacterial genome extraction kit (GeneRay, Shanghai, China) according to the manufacturer’s instructions and subjected to whole-genome sequencing using a 150 bp paired library with the Illumina HiSeq 4000 platform at Novogene Bioinformatics Institute (Beijing, China). A total of 21.96 Gb (90.93% out of 24.15 Gb) of high-quality paired-end reads was retained for further analyses. Raw reads were trimmed and assembled into contigs using CLC Genomics Workbench CLCv.12.0.3 (CLC Bio, Aarhus, Denmark). The genomes were automatically annotated by the NCBI Prokaryotic Genome Annotation Pipeline (PGAP) and deposited into GenBank BioProject PRJNA769251. Identification of acquired antimicrobial resistance genes was conducted using the ResFinder 4.0 databases^1^ (Bortolaia et al., 2020), and mobile genetic elements and their related antimicrobial resistance genes and virulence factors were identified by Mobile Element Finder^2^. Phylogenetic analysis of the genomes was performed by KSNP3.0 software using the maximum-likelihood method, with SNP detection based on k-mer analysis (Sommer, 2015). The pairwise SNP distance for 20 *Lactiplantibacillus* isolates was analyzed by the bioinformatics tool snp-dists 0.7.0 in the Bactopia Analysis Pipeline (Petit and Read, 2020). The whole-genome similarity between *Lactiplantibacillus* isolates was determined by the comparison of average nucleotide identity (ANI) values calculated using JSpecies software (Richter et al., 2016).

### Phenotypic Analysis

To correlate IRBT types with phenotypic attributes, ten *Lactiplantibacillus* isolates (C7-85, C7-7, C7-39, C7-52, R62, R95, R47, R75, R98, and R106), which represented the five clusters, respectively, were further selected for probiotic physiological attributes characterization (Table 1).

#### Artificial Gastric Juice and Intestinal Juice Susceptibility

Gastric juice susceptibility (GJS) and bile salt susceptibility (BSS) were assessed according to previous studies with some modifications (Silva et al., 2013; Liu et al., 2021). *Lactiplantibacillus* cultures in the stationary phase were suspended in 0.9% saline at pH 7.0 (control), artificial gastric juice (NaCl 2 g/L, pepsin 3.2 g/L, pH 2.5) or artificial intestinal juice (NaHCO_3_ 150 mM, trypsin 1.9 g/L, pH 8.0) and incubated at 37°C for 3 h. The samples were centrifuged, and the pellets were suspended in 1 mL MRS broth.

For GJS and BSS analyses, each culture was transferred to an Eppendorf microtube and diluted to 2% (v/v) in either MRS broth or MRS broth supplemented with 0.3% bile acids (Hopebio). Then, 200 μL bacterial suspensions were aliquoted into sterile 96-well microplates and incubated in a thermoregulated spectrophotometer (Microplate Spectrophotometer System SpectraMax i3x, Molecular Devices, Sunnyvale, CA, United States) for 18 h at 37°C. The absorbance was determined by the OD_620_ measured every 30 min. The growth inhibition percentage was calculated using GraphPad Prism 8.0 according to the formula (1–area_*S*_/area_*CT*_) × 100, where area_*S*_ and area_*CT*_ are the areas under the growth curve of the stressed strains (artificial gastric juice or bile salts) and controls, respectively. The strains were classified as resistant at GJS/BSS <40%, moderately resistant at 40% ≤ GJS/BSS ≤ 75% or susceptible at GJS/BSS >75%. The results were based on the average of three independent assays.

#### Surface Hydrophobicity Test

The hydrophobic/hydrophilic cell-surface properties of *Lactiplantibacillus* isolates were assessed by microbial adhesion to solvents (MATS) according to the methodology described by Silva et al. (2013) and Liu et al. (2021). *Lactiplantibacillus* cultures in the stationary phase were centrifuged, washed twice with PBS, and adjusted to an OD_600_ of 0.6 with 0.1 M KNO_3_ at pH 6.2 (A0). Next, 0.4 mL of xylene was mixed with 2.0 mL of each microbial suspension by vortexing for 2 min. The aqueous phase was removed, and the OD_600_ was measured (A1). MATS was calculated according to the formula (1 – A1/A0) × 100. The isolates were classified as hydrophobic at MATS >70%, amphiphilic at 30% ≤ MATS ≤ 70%, or hydrophilic at MATS <30%. The results were based on the average of three independent assays.

#### Antibiotic Susceptibility Test

The antibiotic susceptibility test was performed by the agar disk diffusion method (Sandes et al., 2017). Isolated *Lactiplantibacillus* strains were grown on MRS agar (Oxoid, Basingstoke, United Kingdom) under aerobiosis for 48 h at 37°C. Then, viable cell suspensions at a concentration of 10^8^ (0.5 McFarland scale) were prepared using 0.85% buffered saline and spread onto MRS agar (Oxoid, Basingstoke, United Kingdom). Antibiotic disks were distributed on the surface of agar, and the plates were incubated for around 48 h at 37°C. Then, the diameters of the inhibition zones (mm) were recorded according to automatic inhibition zone measurement with a colony counter (Czone 8; Shineso Science & Technology Co., Ltd., Hangzhou, China). The 15 disks (Oxoid^®^, Basingstoke, United Kingdom) used contained amoxicillin-AMC (30 μg), erythromycin-E (15 μg), clindamycin-DA (2 μg), chloramphenicol-C (30 μg), tetracycline-TE (30 μg), gentamicin-CN (10 μg), ampicillin-AMP (10 μg), sulfamethoxazole-SXT (25 μg), ceftriaxone-CRO (30 μg), kanamycin-K (30 μg), streptomycin-S (10 μg), enrofloxacin-ENR (5 μg), penicillin-G-P (10 U), cefoxitin-FOX (30 μg), and quinupristin-QD (15 μg). Quality control of the antimicrobial disks was performed using *Escherichia coli* ATCC 25922. *Lactiplantibacillus* isolates were classified as resistant, moderately sensitive, or sensitive according to the cutoff levels proposed by Charteris et al. (1998). Aminoglycoside (gentamicin, kanamycin, streptomycin, and neomycin), ciprofloxacin, and trimethoprim antibiotic resistance is considered intrinsic to most *Lactobacillus* species. Besides, lactobacilli are susceptible to penicillin and β-lactams, chloramphenicol, tetracycline, erythromycin, linezolid, and quinupristin–dalfopristin (Campedelli et al., 2019).

### Statistical Analysis

Non-hierarchical clustering (K-means) analysis and principal component analysis (PCA) based on data from gastric juice and bile salt susceptibility, surface hydrophobicity, and antibiotic susceptibility testing of the samples were conducted using R (version 4.0.2) to estimate and classify the probiotic phenotypes of *Lactiplantibacillus* isolates (R Core Team, 2020).

## Results

### IR Biotyper Analysis

**FIGURE 1:**
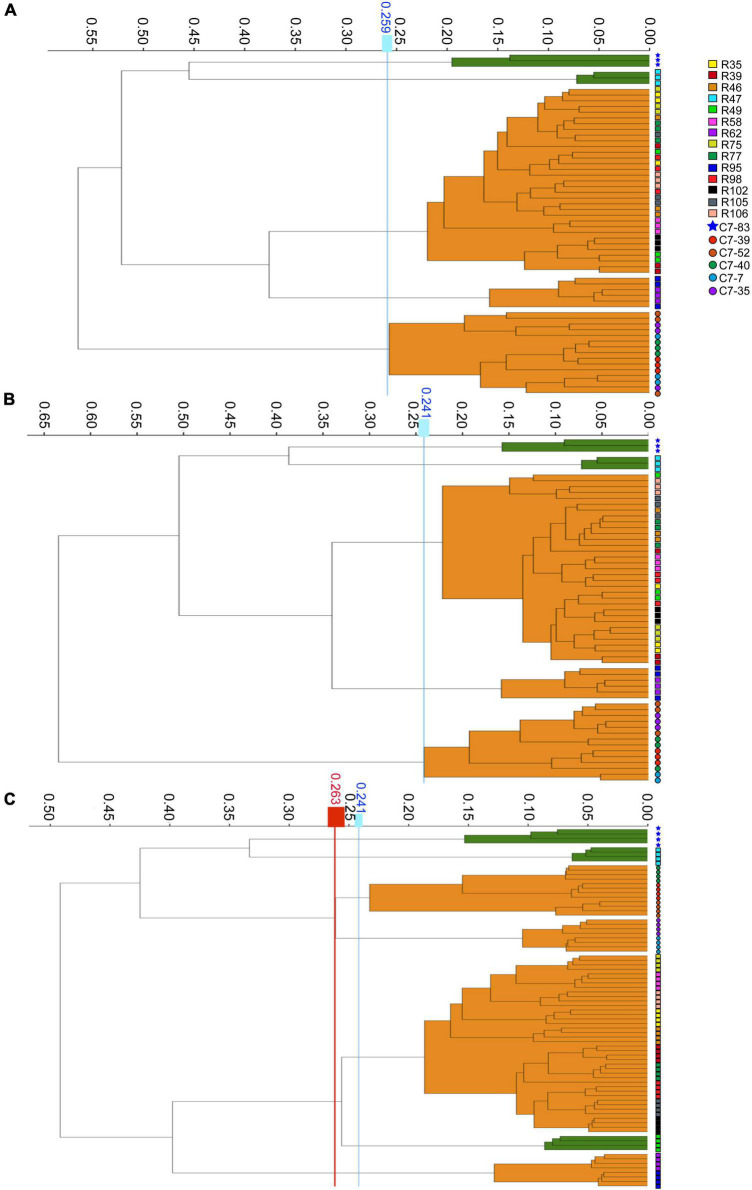
Dendrograms obtained by clustering the IRBT spectra of the twenty *Lactiplantibacillus* isolates. The vertical lines represent the cutoff values. **(A–C)** Represent clusters obtained on three independent days with corrected cutoff-off values of 0.259, 0.241, and 0.263, respectively. The IRBT spectra in **(A,B)** were obtained from the Zhejiang Academy of Agricultural Sciences, and the IRBT spectra in **(C)** were obtained from The Second Affiliated Hospital of Zhejiang University.

The WGS typing results were used as a reference to assess the reproducibility of IRBT. For each dendrogram, the IRBT software automatically calculated a COV. The IRBT spectra in Figures 1A,B from the same laboratory clustered the 20 *Lactiplantibacillus* isolates (three technical replicates) into five different IR types (a∼e) (Supplementary Figure 1), corresponding to five different sequence types (1∼5) according to WGS, while inconsistent with those (four technical replicates) in Figure 1C from a different laboratory. The COV automatically calculated by the IRBT software for Figure 1C was 0.241, which distributed the 20 isolates into seven clusters that were not in agreement with the results of WGS. Therefore, the corrected COV 0.263, which was set on the “right-most” side of the distance between the two nodes (forming a new cluster and a higher distance value for the current nodes), was adopted in our study to avoid such interlaboratory deviation of discriminatory power of IRBT at the strain level. The corrected COVs (ranging from 0.241 to 0.263), all yielded results consistent with those of WGS, indicating good reproducibility and robustness of IRBT.

### Comparison With Other Genotyping Methods

**FIGURE 2:**
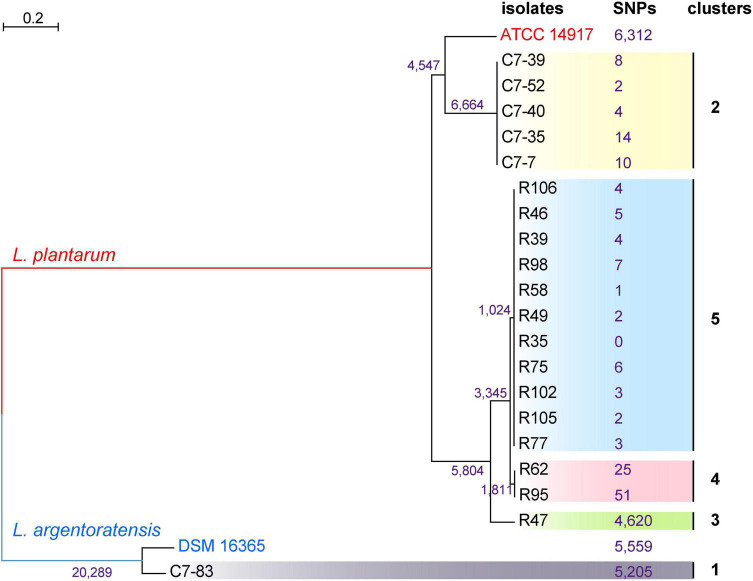
Phylogenetic tree of 20 *Lactiplantibacillus* isolates and two reference strains, *L. argentoratensis* DSM 16365 and *L. plantarum* ATCC 14817 based on whole-genome SNPs, showing five main clades. SNPs that unique to each node identified by the kSNP are marked.

To generate a reference for the spectrum-based typing method, a whole-genome SNP-based phylogenetic comparison of 20 sequenced *Lactiplantibacillus* genomes was conducted. The number of SNPs identified by the kSNP that are unique to each node was annotated in the phylogenetic tree (Figure 2). WGS allowed the assignment of the 20 isolates to 5 lineages. Two WGS clusters contained only a single isolate, C7-83 and R47, whereas the other three clusters comprised 2–11 isolates. Within the same lineage, the maximum SNP differences between two isolates in lineage 4 was 76. Supplementary Table 1 shows the pairwise SNP distance matrix for the 20 isolates via a core-genome alignment. We found that intra-strain SNP threshold obtained by snp-dists was 12, inconsistent with the kSNP results. Therefore, using SNP threshold alone to assess whether bacteria originated from the same source can be misleading.

**FIGURE 3:**
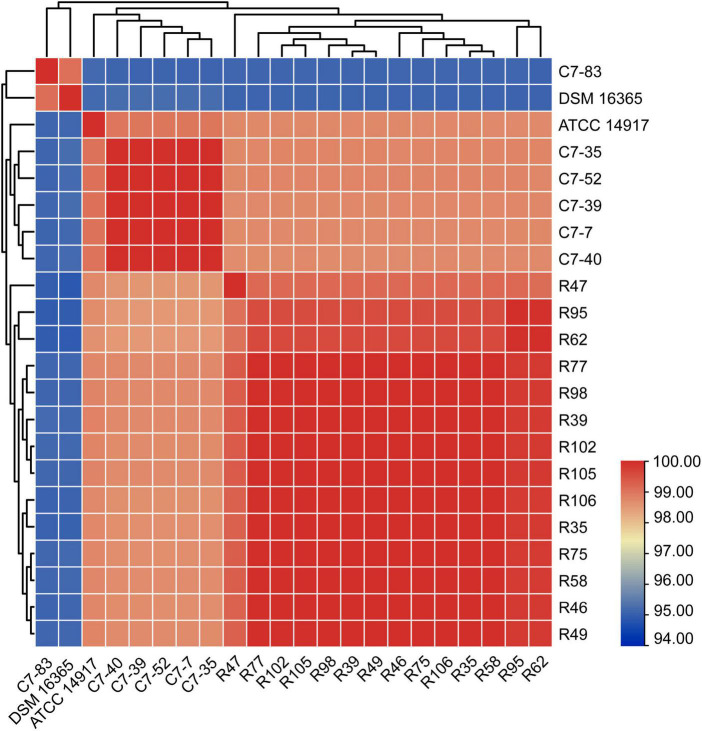
Heatmap of the interspecies genomic similarity based on the % ANI of 20 *Lactiplantibacillus* isolates and two reference strains, *L. argentoratensis* DSM 16365 and *L. plantarum* ATCC 14817. The bar on the right represents the color codes for ANI values from pairwise comparisons of isolates.

The ANI values between the WGS data of each isolate and the reference genomes from two closely related species, *L. plantarum* and *L. argentoratensis*, were calculated (Figure 3). ANI value ≥95% indicates classification as the same species (Richter and Rosselló-Móra, 2009). All isolates showed ANI values >94.8% relative to the two references, with isolate C7-83 and the other 19 isolates displaying ANI values >98.6% relative to the reference strains *L. argentoratensis* DSM 16365 and *L. plantarum* ATCC 14817, respectively. ANI analysis confirmed that isolate C7-83 belongs to *L. argentoratensis*, whereas the other 19 isolates belong to *L. plantarum*. Hereinafter, the species attributions of the isolates used in this study are derived from the ANI calculations.

Comparison of IRBT spectroscopic typing with other genotypic typing results was shown in Table 2. The clustering results of IRBT, WGS, and PFGE were entirely consistent with each other, and all had higher discriminatory power than that of MLST. The MLST method failed to distinguish R62 and R95 from other *L. plantarum* strains.

**TABLE 2:** Comparison of IRBT spectroscopic typing with other genotypic and phenotypic typing results.

					Allele no. at locus							
Isolate	IRBT	WGSST	PFGE	MLST	*pgm*	*ddl*	*gyrB*	*mutS*	*gdh*	*purK1*	*tkt4*	PT
C7-83	a	1	A	i	3*	3*	3*	4*	6*	7*	ND	I
C7-7	b	2	B	ii	1	1	1	1	9	5	1	II
C7-35	b	2	B	ii	1	1	1	1	9	5	1	ND
C7-39	b	2	B	ii	1	1	1	1	9	5	1	II
C7-40	b	2	B	ii	1	1	1	1	9	5	1	ND
C7-52	b	2	B	ii	1	1	1	1	9	5	1	II
R47	c	3	C	iii	1	1*	4*	1*	1*	3*	4*	III
R62	d	4	D	iv	2*	1*	4*	1*	10*	3	4*	IV
R95	d	4	D	iv	2*	1*	4*	1*	10*	3	4*	IV
R35	e	5	E	iv	2*	1*	4*	1*	10*	3	4*	ND
R39	e	5	E	iv	2*	1*	4*	1*	10*	3	4*	ND
R46	e	5	E	iv	2*	1*	4*	1*	10*	3	4*	ND
R49	e	5	E	iv	2*	1*	4*	1*	10*	3	4*	ND
R58	e	5	E	iv	2*	1*	4*	1*	10*	3	4*	ND
R75	e	5	E	iv	2*	1*	4*	1*	10*	3	4*	V
R77	e	5	E	iv	2*	1*	4*	1*	10*	3	4*	ND
R98	e	5	E	iv	2*	1*	4*	1*	10*	3	4*	V
R102	e	5	E	iv	2*	1*	4*	1*	10*	3	4*	ND
R105	e	5	E	iv	2*	1*	4*	1*	10*	3	4*	ND
R106	e	5	E	iv	2*	1*	4*	1*	10*	3	4*	V

*Represents the closest variant to the corresponding locus, with a 1–6 SNPs; ND, no data available. Each allele was assigned a different allele number, and the allelic profile (string of seven integers) was defined by combining the numbers corresponding to alleles at the loci analyzed. IRBT, IR Biotyper; WGS, whole-genome sequencing; ST, sequence types; PFGE, pulsed-field gel electrophoresis; MLST, multilocus sequence typing; AP, allelic profiles; PT, phenotypic typing.

### Concordance With Physiological Attributes

**FIGURE 4:**
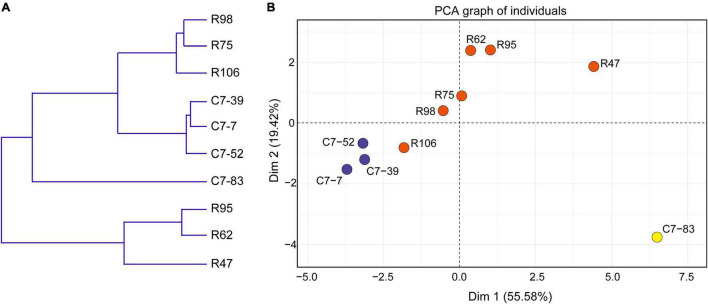
**(A)** Dendrogram of nonhierarchical clustering analysis and **(B)** score plot of principal component analysis for the ten *Lactiplantibacillus* isolates based on probiotic physiological data. Axis 1 and 2 accounted for 55.6% and 19.4% of the total variation present, respectively.

Data from the 10 isolates regarding the phenotypes (acid and bile acid susceptibility, surface hydrophobicity, and antibiotic susceptibility) that associate with *in vitro* probiotic screening (Supplementary Tables 2, 3) were clustered and subjected to PCA analysis. As illustrated in Figure 4A, clustering analysis classified the 10 isolates into three major clusters and five subclusters (I–V). In accordance with the IRBT and genomic results, isolates C7-85 and R47 alone formed clusters I and III, respectively. Isolates R62 and R95 formed cluster IV. Cluster II included isolates C7-7, C7-39, and C7-52, whereas Cluster V contained isolates R75, R98, and R106.

Furthermore, based on various physiological properties relevant to probiotic safety, gastrointestinal viability, and persistence, PCA was performed to assess the variability and similarities among the 10 isolates. As illustrated in Figure 4B, the first and second principal components represented 55.6 and 19.4% of the total 18 variables (GJS, BSS, MAST, and the diameters of the bacteriostatic circles of 15 antibiotics), respectively, and indicated the significant role of gastrointestinal tolerance proprieties in probiotic screening. Clear visual separation of the 10 isolates into five point sets was observed in the PCA plot, with isolate C7-83 being the most distant from the other isolates. These results indicated that differences in IRBT spectra can be reflected in probiotic related phenotypes and that the typing results from genotypic, phenotypic, and spectroscopic analyses were consistent (Table 2).

## Discussion

To date, successful IRBT typing of probiotics at the strain level has only been reported for *Bifidobacterium* (Deidda et al., 2021). In lactic acid bacteria, only species-level typing of *lactobacilli* by FTIR has been reported (Oust et al., 2004; Bosch et al., 2006; Santos et al., 2015; Quintelas et al., 2018). The potential application of IRBT in probiotic industries has yet to be explored. The present study was the first to evaluate the ability of IRBT to type *L. plantarum* at the strain level. IRBT successfully distinguished the two closely related species within the *Lactiplantibacillus* genus, namely, *L. plantarum* and *L. argentoratensis*. Surprisingly, it also has potential to discriminate the different strains belonging to *L. plantarum*. The current definition of “strain” is confusing, and the genetic SNP thresholds for strain delineation have not been universally set in culture-centric microbiology. A “taxonomic strain” is defined as the descendants of a single isolation in pure culture and usually consisting of a succession of cultures ultimately derived from an initial single colony, whereas a “natural strain” is defined as a set of conspecific isolates with distinctive genotypic and/or phenotypic characteristics. Theoretically, it is possible to distinguish two “natural strains” based on a single nucleotide difference, even if no phenotypic differences are identified. By contrast, “taxonomic strains” can become phenotypically heterogeneous with as few as three mutations, but would still be called the same strain (Jackson et al., 2019; Van Rossum et al., 2020). Our study found that 20 *L. plantarum* isolates were consistently grouped into five IRBT spectral types, corresponding to five different WGS lineages, and five phenotypic patterns associated with probiotic screening.

Microorganisms, including probiotic, pathogenic or neutral strains, play a vital role in human health. On the one hand, certain pathogenic bacteria can cause a series of diseases after invading the host. On the other hand, symbiotic bacteria establish a mutually beneficial relationship with the host. Some probiotics may become adjuvant therapies in the treatment of certain diseases (Zhu and Liu, 2017; Atefi et al., 2021; Xu et al., 2021). Therefore, typing pathogenic and probiotic bacteria at the strain level is a tremendous challenge relevant to human health. However, in the field of microbial typing, IRBT is mainly used in routine clinical hygiene and infection control. The typing function of FTIR has been verified in a large number of gram-negative bacteria in the clinic, such as *E. coli*, *Klebsiella oxytoca* and *Yersinia enterocolitica*, as well as isolates that can cause outbreaks in hospitals such as *Enterobacter cloacae*, *Klebsiella pneumoniae* and *Pseudomonas aeruginosa* (Dieckmann et al., 2016; Dinkelacker et al., 2018; Quintelas et al., 2018; Martak et al., 2019; Novais et al., 2019; Hu et al., 2020). For gram-positive bacteria, the strain-level distinguishing ability of FTIR has been extensively explored in species that are mainly considered relevant to human diseases or food safety, such as *Staphylococcus aureus* and *Listeria monocytogenes* (Novais et al., 2019). However, it is necessary to consider not only the harmful factors of pathogenic bacteria but also the positive effects of probiotics. The quality and safety assessment of probiotics in foods and supplements is the responsibility of probiotic industries (Deidda et al., 2021). Due to the growing demand for probiotics, industries need to identify specific probiotics at the strain level quickly and accurately. Considering that the health benefits of probiotics are strain-specific, the European Food Safety Authority also requires the identification of probiotics at the strain level, which provides the basis for health claims (Scientific Opinion on the substantiation of health claims related to non characterised microorganisms pursuant to Article 13(1) of Regulation (EC) No 1924/2006, 2009). In clinical application scenarios, it is also important to consider both probiotic strain specificity and disease specificity to identify the appropriate probiotics that match specific diseases (McFarland et al., 2018). Therefore, the expanded application of IRBT in probiotic industries is urgently needed and necessary.

*Lactiplantibacillus plantarum* is a model species for research in lactobacilli, which comprise an important group of probiotics for both humans and animals (Martino et al., 2016; Choi et al., 2018; Teame et al., 2020; Yu et al., 2020, 2021). *L. plantarum* strains have diverse phenotypes and genotypes that facilitate a metabolic flexibility that allow them to colonize a variety of environments, including the human gastrointestinal tract (Pretzer et al., 2005; Marco et al., 2010; Fidanza et al., 2021). There have been reports indicating their potential beneficial effects on humans (Molenaar et al., 2005; Klarin et al., 2008; Siezen et al., 2010; Lewis-Mikhael et al., 2020; Zhao et al., 2021). Its excellent adaptability, extensive industrial applicability and powerful influence on human and animal physiology have made *L. plantarum* a microorganism of great interest to the academic community. Hence, we chose *L. plantarum* as the model bacterium to carry out the strain-level typing evaluation of IRBT, which will have great academic and industrial value.

The integration of hardware and software in IRBT provides a potential turn-key solution for routine use in probiotic industries. Determining a reference COV range is important in implementing IRBT as a new phenotypic typing method for routine use in probiotic typing and screening. The automatic COV is the compromise between finding as many clusters as possible that are pure and that a label is represented only by one cluster (Deidda et al., 2021). IRBT spectroscopy is known to be very sensitive to changes in culture medium, incubation time, temperature, and humidity. Different culture parameters will lead to distinct growth conditions among strains, which will have a significant impact on IRBT clustering results (Hu et al., 2020). In this research, fixed culture conditions for *L. plantarum* were used to obtain robust IRBT typing results within the same laboratory. However, upon introducing spectra from different laboratories, a higher COV and more branches of the clustering dendrogram for *L. plantarum* were observed when automatically calculated by the IRBT software. The deviation of IRBT clustering results across laboratories may have been caused by technical variances rather than biological variances for the following reasons: the frequency of spontaneous mutations in *L. plantarum* is lower than 10^–7^ mutants per plated cell (Nicoloff et al., 2005). Since strain transfer from one institution to another was controlled within three generations of bacterial growth, mutational events may have occurred in the spontaneous mutants even though this is unlikely. In addition, the IRBT spectroscopy of a particular strain is likely to remain consistent even with few numbers of mutations, for mutations in genes do not necessarily cause epigenetic changes; For reproducibility testing, more technical replicates of each isolate were conducted in the external than internal laboratories. Since the automatic COV tends to find as many clusters as possible (default algorithm of IRBT software), it may underestimate the original COV of the external laboratory, resulting in more clusters being generated than the internal laboratory; It is also worth noting that repeated experiments in different laboratories were not performed by the same technician. In fact, the incubation and preparation practices often have a much greater impact on the results than the drying of the same suspension on different spots. So, it is totally normal that different measurements of the same isolate are distributed farther than technical replica; Furthermore, subtle distinctions in IRBT device sensitivities could not be ruled out, although HCA analysis using Euclidean average linkage as exploration method was selected for all repeated experiments.

Previous studies have shown that various phenotypic and genotypic methods, such as MLST, *Asc* I-PFGE, and WGS, allow *L. plantarum* to be typed at the interspecies and intraspecies levels (Guidone et al., 2014; Martino et al., 2016; Evanovich et al., 2019; Manzoor and Tayyeb, 2019; Yu et al., 2021). Although these methods have completely updated our understanding of the bacterial population structure, they ignore the phenotypic variations caused by certain genetic changes, especially genotypic or phenotypic characteristics related to key macromolecules on the cell surface. In addition, genotyping techniques are usually time-consuming, labor-intensive, and expensive, limiting their wide application. In addition to DNA-based methods, IRBT has recently shown promising results for typing certain groups of gram-positive and gram-negative bacteria (Hu et al., 2020; Deidda et al., 2021). Comparison results in the present study demonstrated that IRBT had higher discriminatory power than MLST and had equivalent discriminatory power to that of the previous and new gold standards, PFGE and WGS, respectively. The MLST analysis failed to differentiate WGS sequence type 4 from 5 may have been due to the selection of insufficient numbers of alleles or inappropriate loci when performing MLST (Yu et al., 2020). In a typing study of *Bifidobacteria*, it is also proven that the resolution of IRBT is better than that of MLST, as IRBT could distinguish all strains of *B. animalis* subsp. *lactis*, while MLST fails to achieve this effect (Deidda et al., 2021).

Selecting probiotics from a large pool of isolates using a series of *in vitro* preliminary selections and followed by *in vivo* validation studies is laborious, expensive, time-consuming, and not easily achievable. *In vitro* selection is therefore the first approach used to select a few strains that can be further evaluated *in vivo* (Morelli, 2000). Due to the diverse ecological niches, phenotypes, and genotypes of *L. plantarum*, a large number of duplicates of the same strain may be isolated from the same niche, and obtaining new isolates from different habitats may also lead to multiple isolations of the same strain (Deidda et al., 2021). This makes the rigorous screening of probiotics from *L. plantarum* isolates very challenging. Therefore, it is particularly necessary to apply a fast, low-cost, and effective technology that can distinguish *L. plantarum* at the strain level to reduce the number of isolates to be considered before performing extensive screening and evaluation processes. PFGE is very laborious, whereas WGS is quite expensive, and both are time-consuming (2–3 days), especially when typing a large group of new isolates. Their applications in routine probiotic identification and typing may be restricted (Novais et al., 2019). In contrast, IRBT is a fast (within 3 h), inexpensive and high-throughput bacterial typing system. Moreover, the spectral typing from IRBT matched the probiotic phenotypic clustering results of the 10 representative *Lactiplantibacillus* isolates. Under the default IRBT settings, the observed spectral range focuses on the polysaccharide region (1,200–900 cm^–1^), which highlights changes in the carbohydrate composition of bacterial walls. These features may be closely related to acid and bile salt sensitivity, cell surface hydrophobicity, and antibiotic resistance, which are related to probiotic phenotypes (Kuda et al., 2013). Several studies have reported that the resistance of *L. plantarum* to acid, heat and other stresses is associated with cell membrane functions (Capozzi et al., 2011; Ricciardi et al., 2012). Therefore, due to the resulting reduced workload, probiotic screening efficiency would be greatly improved by the inclusion of IRBT before performing extensive preliminary selection processes. Based on our IRBT results in *L. plantarum* typing, a good database can be constructed for introduction into the routine procedures of probiotic screening and typing. One of the limitations of our study is the lack of inclusion of the type strain in IRBT, PFGE, and phenotypic studies. *L. plantarum* isolate R106 (CGMCC 20370) could be used for other researchers as a control in such studies.

## Conclusion

In this study, we conducted the first investigation of *L. plantarum* strain typing based on IRBT. We demonstrated that IRBT could successfully strain-level type of *L. plantarum*, which has traditionally been used as a probiotic. A deviation in the discriminative power of IRBT was found interlaboratory at the strain level, probably caused by technical variance. Compared with other genotyping techniques, IRBT was equivalent to the previous and current gold standards, PFGE and WGS, in typing at the strain level and had higher discriminatory power than that of MLST. In the comparison with probiotic screening associated phenotyping, the clustering results of IRBT spectral types and phenotypes were consistent, revealing that these two attributes were closely related. Therefore, the typing results from spectroscopic, genotypic, and probiotic screening related phenotypic analyses were concordant. In addition, thanks to the great advantages of IRBT in DNA-based technology, such as ease of use, fast turnaround time, user-friendly software, and relatively low operating costs, IRBT is suitable for daily strain typing in probiotic laboratories and industries. IRBT not only could effectively improve the probiotic screening efficiency, but also has application potential in the traceability and quality control of probiotics. It is worth noting that the exchange of results between different laboratories based on IRBT requires a high level of standardization.

## Data Availability Statement

The datasets presented in this study can be found in the BioProject PRJNA769251 at DDBJ/ENA/GenBank database. The names of the repository/repositories and accession number(s) can be found in the article/Supplementary Material.

## Author Contributions

XL: conceptualization, writing – original draft, review, and editing, methodology, funding acquisition, and sources. BT: conceptualization, investigation, formal analysis, visualization, and writing – review and editing. JL: project administration, funding acquisition, and supervision. LZ and XW: methodology, investigation, and visualization. All authors contributed to the article and approved the submitted version.

## Conflict of Interest

The authors declare that the research was conducted in the absence of any commercial or financial relationships that could be construed as a potential conflict of interest.

## Publisher’s Note

All claims expressed in this article are solely those of the authors and do not necessarily represent those of their affiliated organizations, or those of the publisher, the editors and the reviewers. Any product that may be evaluated in this article, or claim that may be made by its manufacturer, is not guaranteed or endorsed by the publisher.

## Footnotes

1
https://cge.cbs.dtu.dk/services/ResFinder/

2
https://cge.cbs.dtu.dk/services/MobileElementFinder/
