# Supplementary material for: Evaluation of IR Biotyper for Lactiplantibacillus plantarum Typing and Its Application Potential in Probiotic Preliminary Screening
Source: Front Microbiol. 2022 Mar 24;13:823120. doi: 10.3389/fmicb.2022.823120 (PMC8988154; doi:10.3389/fmicb.2022.823120)
Supplement: Supplementary file 1 [file Data_Sheet_1.docx]

**Supplemental Materials**

**Evaluation of IR Biotyper for *Lactiplantibacillus plantarum* typing and its application potential in probiotic preliminary screening**

Xiaoqiong Li^1^, Liying Zhu^1^, Xin Wang^1^, Jinjun Li^1^*, Biao Tang^2^*

1 State Key Laboratory for Managing Biotic and Chemical Threats to the Quality and Safety of Agro-products and Institute of Food Sciences, Zhejiang Academy of Agricultural Sciences, Hangzhou 310021, China;

2 Institute of Agro-product Safety and Nutrition, Zhejiang Academy of Agricultural Sciences, Hangzhou 310021, China;

*Corresponding:

Jinjun Li (lijinjun@zaas.ac.cn)

Institute of Food Sciences, Zhejiang Academy of Agricultural Sciences, Hangzhou，China;

Biao Tang (tb_411@163.com)

Institute of Agro-product Safety and Nutrition, Zhejiang Academy of Agricultural Sciences, Hangzhou, China

**1. Materials and methods**

**Pulsed-Field Gel Electrophoresis (PFGE) Analysis**

Agarose plugs for PFGE analysis containing intact DNA from the *Lactiplantibacillus* isolates were prepared and treated with suitable endonucleases as reported by(Guidone et al., 2014). Briefly, the DNA was digested for 4.5 h at 37 °C with 30 U of AscI (New England BioLabs, Inc., Ipswich, MA) in 150 μL of the supplied restriction buffer. *Salmonella enterica* serotype *Braenderup* strain H9812 digested with restriction enzyme *Xba* I (New England BioLabs, USA) was used as a size marker. The gel used for PFGE analysis contained 1% (w/v) SeaKem Gold agarose (Lonza, USA) in 0.5× Tris–Borate–EDTA buffer. PFGE was performed on a CHEF-mapper system (Bio–Rad Laboratories, USA) for 20 h at 6 V/cm and a temperature of 14 °C. The fragments from the isolates were run at pulses from 1 to 15 s. After staining with Gelred, images were obtained using a Bio–Rad Gel Doc XR system with Quantity One 4.4.1 software (Bio–Rad Laboratories, USA). Image processing and clustering analysis of PFGE patterns were performed using BioNumerics 7.6 software (Applied Maths, Saint-Matins-Latem, Belgium).

**Multilocus Sequence Typing (MLST) Analysis**

The sequences of seven housekeeping genes (phosphoglucomutase (*pgm*), D-alanine-D-alanine ligase (*ddl*), the B subunit of DNA gyrase (*gyrB*), the ATPase subunit of phosphoribosylami-noimidazole carboxylase (*purK1*), glutamate dehydrogenase (*gdh*), DNA mismatch repair protein (*mutS*) and transketolase (*tkt4*)) included in the MLST analyses were extracted from WGS data. Allelic profiles were determined by the MLST method described by de las Rivas et al. (2006). The sequences obtained for all isolates were compared, and allele numbers were assigned to each unique sequence. Each isolate was defined by an allelic profile derived from the combination of numbers corresponding to alleles at the analyzed loci (**Table 2**). Sequences that differed at a single nucleotide site were considered distinct alleles.

**2. Results and Discussion**

**2.1 PFGE**

The isolates were subtyped by PFGE, and a dendrogram based on their similarities was generated (**Fig. S2**). AscI-digested *Lactiplantibacillus* DNA generated 5 unique PFGE patterns (A-E). PFGE Patterns B, D and E were represented by more than one isolate, with pattern E (n = 11) containing the most isolates, followed by pattern B (n = 5). In fact, C7-83, which was the only strain belonging to *L.* *argentoratensis*, showed a discernible pattern compared with the other isolates. In addition, most of the isolates collected from the same fermentation day were closely clustered in accordance with the time origin in the dendrogram.

**2.2 MLST**

The allelic profiles (APs) of each isolate are shown in (**Fig. S3, Table 2**). Since the *tkt4* allele sequence of the C7-83 isolate was not available, it was excluded from MLST analysis. MLST analysis based on six loci (which were extracted from WGS data) differentiated the 20 isolates into 4 APs (i - ⅳ). All APs differed in at least 2 loci. Compared with PFGE and SNP-based WGS, the MLST method failed to distinguish R62 and R95 from other *L. plantarum* strains, and its resolution was lower than those of other genotyping methods.

**2.3 Probiotic *in vitro* screening**

**2.3.1 Gastric Juice and Bile Salt Susceptibility**

Probiotic candidates need to be able to survive in the gastrointestinal tracts of their hosts to exert beneficial effects; therefore, we evaluated the isolate susceptibility to gastric juice and bile salts. **Table S2** shows the GJS and BSS values of the 10 tested isolates. Of these isolates, only 3 isolates, C7-85, C7-7 and C7-39, were identified as resistant to low pH and bile salts (GJS/BSS <40%). Six isolates, R62, R95, R47, R75, R98 and R106, showed resistance to acid and moderate resistance to bile salts (GJS <40%, 40 ≤ BSS ≤ 75%). The only *L. argentoratensis* strain, C7-83, was sensitive to acid and bile salts (GJS/BSS >75%). Notably, five types of growth pattern were observed of 10 representative *Lactiplantibacillu*s isolates in MRS broth at 37 °C with pH = 7.4 or pH = 2.5 or in 0.3% bile acids for 18 h (**Fig. S4**)

**2.3.2 Surface Hydrophobicity**

The ability of bacteria to adhere to apolar surfaces such as the mucosal epithelium of the gut can be indirectly indicated by surface hydrophobicity. The surface hydrophobicity of the 10 *Lactiplantibacillus* isolates varied from 2.3% to 86.4% (**Table S2**). The surface hydrophobicity of isolates R62 and R95 (IRBT-d), which were classified as hydrophobic (MAST > 70%), was higher than those of the other strains. Isolates C7-85 (IRBT-a), C7-7, C7-39 and C7-52 (IRBT-b), R75, R98 and R106 (IRBT-e), which were classified as hydrophilic (MAST < 30%), exhibited relatively low hydrophobicity, whereas isolate R47 (IRBT-c), with a MAST value of 54.5%, was regarded as amphiphilic.

**2.3.3 Antibiotics Susceptibility**

The antimicrobial resistance results of the 10 *Lactiplantibacillus* isolates for different antibiotics are shown in **Table S3**. All 10 isolates exhibited resistance to at least one of the 15 antibiotics tested. Strain C7-85 exhibited resistance to enrofloxacin (one), R47 exhibited resistance to enrofloxacin and moderate sensitivity to streptomycin (two), R62 and R95 exhibited resistance to kanamycin and gentamicin in addition to enrofloxacin and streptomycin (four), while the remaining strains exhibited multiple-drug resistance. All strains were sensitive to 10 antibiotics, namely, amoxicillin, erythromycin, clindamycin, chloramphenicol, tetracycline, ampicillin, cefatriaxone, sulfamethoxazole, penicillin-G, cefoxitin and quinupristin. None of the strains had acquired antibiotic-resistance genes on their genomes, indicating that these isolates were safe for future application as probiotics.

**References**

de las Rivas, B., Marcobal, Á., and Muñoz, R. (2006). Development of a multilocus sequence typing method for analysis of *Lactobacillus plantarum* strains. *Microbiology*. doi:10.1099/mic.0.28482-0.

Guidone, A., Zotta, T., Ross, R. P., Stanton, C., Rea, M. C., Parente, E., et al. (2014). Functional properties of *Lactobacillus plantarum* strains: A multivariate screening study. *LWT - Food Sci. Technol.* doi:10.1016/j.lwt.2013.10.036.

**Table S1.** Pairwise SNP distance matrix for 20 *Lactiplantibacillus* isolates analyzed by the bioinformatics tool snp-dists 0.7.0 in the Bactopia Analysis Pipeline.

|  | **C7-35** | **C7-39** | **C7-40** | **C7-52** | **C7-7** | **C7-83** | **R102** | **R105** | **R106** | **R35** | **R39** | **R46** | **R47** | **R49** | **R58** | **R62** | **R75** | **R77** | **R95** | **R98** |
| --- | --- | --- | --- | --- | --- | --- | --- | --- | --- | --- | --- | --- | --- | --- | --- | --- | --- | --- | --- | --- |
| **C7-35** | 0 | 1 | 1 | 1 | 1 | 64278 | 11403 | 11402 | 11403 | 11403 | 11405 | 11403 | 11561 | 11403 | 11403 | 11413 | 11405 | 11404 | 11419 | 11404 |
| **C7-39** | 1 | 0 | 0 | 0 | 0 | 64278 | 11402 | 11401 | 11402 | 11402 | 11404 | 11402 | 11560 | 11402 | 11402 | 11412 | 11404 | 11403 | 11418 | 11403 |
| **C7-40** | 1 | 0 | 0 | 0 | 0 | 64278 | 11402 | 11401 | 11402 | 11402 | 11404 | 11402 | 11560 | 11402 | 11402 | 11412 | 11404 | 11403 | 11418 | 11403 |
| **C7-52** | 1 | 0 | 0 | 0 | 0 | 64278 | 11402 | 11401 | 11402 | 11402 | 11404 | 11402 | 11560 | 11402 | 11402 | 11412 | 11404 | 11403 | 11418 | 11403 |
| **C7-7** | 1 | 0 | 0 | 0 | 0 | 64278 | 11402 | 11401 | 11402 | 11402 | 11404 | 11402 | 11560 | 11402 | 11402 | 11412 | 11404 | 11403 | 11418 | 11403 |
| **C7-83** | 64278 | 64278 | 64278 | 64278 | 64278 | 0 | 65195 | 65194 | 65193 | 65193 | 65195 | 65193 | 65435 | 65193 | 65193 | 65189 | 65195 | 65194 | 65195 | 65194 |
| **R102** | 11403 | 11402 | 11402 | 11402 | 11402 | 65195 | 0 | 1 | 2 | 2 | 4 | 2 | 4020 | 2 | 2 | 428 | 4 | 3 | 440 | 3 |
| **R105** | 11402 | 11401 | 11401 | 11401 | 11401 | 65194 | 1 | 0 | 1 | 1 | 3 | 1 | 4019 | 1 | 1 | 427 | 3 | 2 | 439 | 2 |
| **R106** | 11403 | 11402 | 11402 | 11402 | 11402 | 65193 | 2 | 1 | 0 | 0 | 2 | 0 | 4018 | 0 | 0 | 426 | 2 | 1 | 438 | 1 |
| **R35** | 11403 | 11402 | 11402 | 11402 | 11402 | 65193 | 2 | 1 | 0 | 0 | 2 | 0 | 4018 | 0 | 0 | 426 | 2 | 1 | 438 | 1 |
| **R39** | 11405 | 11404 | 11404 | 11404 | 11404 | 65195 | 4 | 3 | 2 | 2 | 0 | 2 | 4020 | 2 | 2 | 428 | 4 | 3 | 440 | 3 |
| **R46** | 11403 | 11402 | 11402 | 11402 | 11402 | 65193 | 2 | 1 | 0 | 0 | 2 | 0 | 4018 | 0 | 0 | 426 | 2 | 1 | 438 | 1 |
| **R47** | 11561 | 11560 | 11560 | 11560 | 11560 | 65435 | 4020 | 4019 | 4018 | 4018 | 4020 | 4018 | 0 | 4018 | 4018 | 4023 | 4020 | 4019 | 4025 | 4019 |
| **R49** | 11403 | 11402 | 11402 | 11402 | 11402 | 65193 | 2 | 1 | 0 | 0 | 2 | 0 | 4018 | 0 | 0 | 426 | 2 | 1 | 438 | 1 |
| **R58** | 11403 | 11402 | 11402 | 11402 | 11402 | 65193 | 2 | 1 | 0 | 0 | 2 | 0 | 4018 | 0 | 0 | 426 | 2 | 1 | 438 | 1 |
| **R62** | 11413 | 11412 | 11412 | 11412 | 11412 | 65189 | 428 | 427 | 426 | 426 | 428 | 426 | 4023 | 426 | 426 | 0 | 428 | 427 | 12 | 427 |
| **R75** | 11405 | 11404 | 11404 | 11404 | 11404 | 65195 | 4 | 3 | 2 | 2 | 4 | 2 | 4020 | 2 | 2 | 428 | 0 | 3 | 440 | 3 |
| **R77** | 11404 | 11403 | 11403 | 11403 | 11403 | 65194 | 3 | 2 | 1 | 1 | 3 | 1 | 4019 | 1 | 1 | 427 | 3 | 0 | 439 | 2 |
| **R95** | 11419 | 11418 | 11418 | 11418 | 11418 | 65195 | 440 | 439 | 438 | 438 | 440 | 438 | 4025 | 438 | 438 | 12 | 440 | 439 | 0 | 439 |
| **R98** | 11404 | 11403 | 11403 | 11403 | 11403 | 65194 | 3 | 2 | 1 | 1 | 3 | 1 | 4019 | 1 | 1 | 427 | 3 | 2 | 439 | 0 |

**Table S2.** Susceptibility to artificial gastric fluid and bile salts and surface hydrophobicity of 10 representative *Lactiplantibacillus* isolates

|  | C7-83 | R47 | R62 | R95 | R75 | R98 | R106 | C7-7 | C7-39 | C7-52 |
| --- | --- | --- | --- | --- | --- | --- | --- | --- | --- | --- |
| GJS (%) | 75.3 | -22.8 | -1.7 | 7.4 | 6.8 | 12.9 | 5.4 | -0.6 | 6.1 | -0.4 |
| BSS (%) | 83.6 | 67.6 | 71.5 | 74.1 | 64.6 | 66.5 | 60.9 | 36.9 | 34.6 | 31.9 |
| MAST (%) | 21.2 | 54.5 | 86.6 | ***74.6*** | 9.7 | 2.3 | 14.1 | 15.0 | 12.0 | 10.0 |

| S | >75% | MR | 40%-75% | R | <40% |
| --- | --- | --- | --- | --- | --- |
| HI | <30% | AI | 30%-70% | HO | >70% |

S: susceptible to GJS/BSS; MR: moderately resistant to GJS/BSS; R: resistant to GJS/BSS; HI: hydrophilic; AI: amphiphilic; HO: hydrophobic. GJS: gastric juice susceptibility; BSS: bile salt susceptibility; MAST: microbial adhesion to solvents. Data are expressed as the mean±standard deviation (n=3). Different physiological phenotypes are marked by different background colors, and misclassed numbers are written in **bold and italics**.

**Table S3.** Resistance to 15 different antibiotics of 10 representative *Lactiplantibacillus* isolates

| Antibiotics | Inhibition zone diameter (mm) | | | | | | | | | |
| --- | --- | --- | --- | --- | --- | --- | --- | --- | --- | --- |
|  | C7-83 | R47 | R62 | R95 | R75 | R98 | R106 | C7-7 | C7-39 | C7-52 |
| Amoxicillin | 48.1 | 43.6 | 43.5 | 45.8 | 45.2 | 44.6 | 43.4 | 41.47 | 41.5 | 42.2 |
| Erythomycin | 33.9 | 37 | 30.2 | 33.7 | 33.7 | 32 | 29.8 | 27.9 | 28.9 | 30 |
| Clindamycin | 20.4 | 31.6 | 23.4 | 27.9 | 41.9 | 39 | 33.5 | 24.1 | 25.4 | 26.9 |
| Chloramphenicol | 24 | 33 | 34.4 | 32.8 | 35.6 | 31.8 | 29.5 | 33.2 | 32.7 | 33.5 |
| Tetracycline | 34.4 | 30.1 | 28 | 26 | 28.4 | 25.3 | 25.7 | 26.9 | 25.5 | 25.1 |
| Gentamicin | 26.5 | 18.9 | 12.7 | 13.8 | 14.1 | ***12.0*** | ***11.2*** | 9.9 | 10.4 | 9.5 |
| Ampicillin | 42.7 | 52 | 45.2 | 46 | 44.7 | 41.6 | 34.6 | 34.5 | 37.5 | 40.5 |
| Sulfamethoxazole | 35.2 | 34.6 | 26 | 25.8 | 27.1 | 27.9 | 26.1 | 25.2 | 23.1 | 25.8 |
| Cefatriaxone | 42.8 | 43.6 | 45.8 | 46 | 42.7 | 41.9 | 42 | 33.1 | 36.1 | 36 |
| Kanamycin | 22.2 | 20.3 | - | - | - | - | - | - | - | - |
| Streptomycin | 23.6 | 13.7 | - | - | - | - | - | - | - | - |
| Enrofloxacin | - | - | 8.5 | 9.1 | 0 | 9.1 | 8.3 | 9.2 | - | 8.7 |
| Penicillin-G | 37.2 | 40.1 | 38.8 | 40 | 34.6 | 35.9 | 30.7 | 27.2 | 30.4 | 26.9 |
| Cefoxitin | 24.2 | 32.1 | 26.3 | 24.5 | 17.9 | 17.8 | 14.8 | 17.5 | 17.1 | ***18.4*** |
| Quinupristin | 36 | 35.1 | 31.4 | 31.7 | 30.8 | 32.4 | 31 | 28.5 | 28.2 | 29 |

| R | < 10 mm | I | 10-20 mm | S | >20 mm |  |
| --- | --- | --- | --- | --- | --- | --- |

"-" represents a noninhibitory circle. R: resistant to antibiotics; I: intermediate resistance to antibiotics; S: sensitive to antibiotics. Data are expressed as the mean (*n*=3). Different physiological phenotypes are marked by different background colors, and misclassed numbers are written in **bold and italics**. The absence of a growth inhibition zone around an antimicrobial discs is described as bacterial resistance.


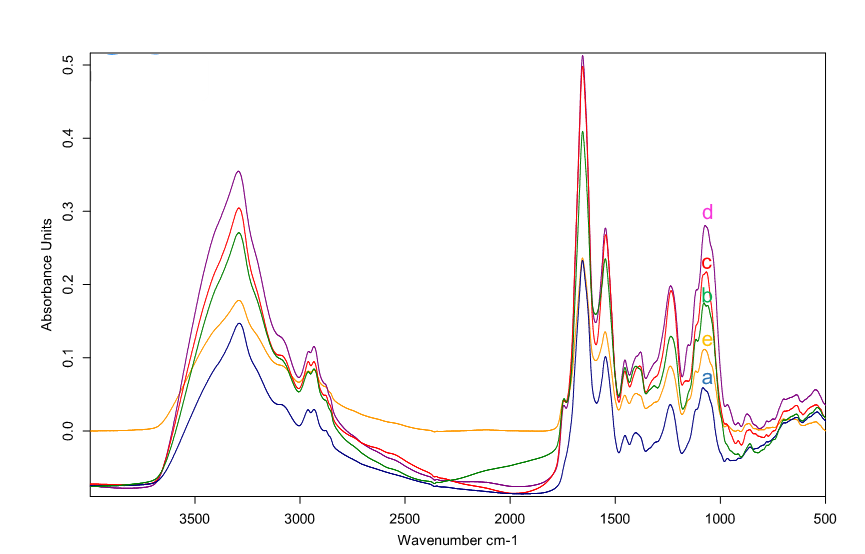


**Figure S1**. Five representative IRBT spectra of *Lactiplantibacillus* (4000 to 500 cm^-1^), in which a ~ e represent 5 different strains, C7-83, C7-7, R47, R95 and R106, respectively, with specific probiotic properties.


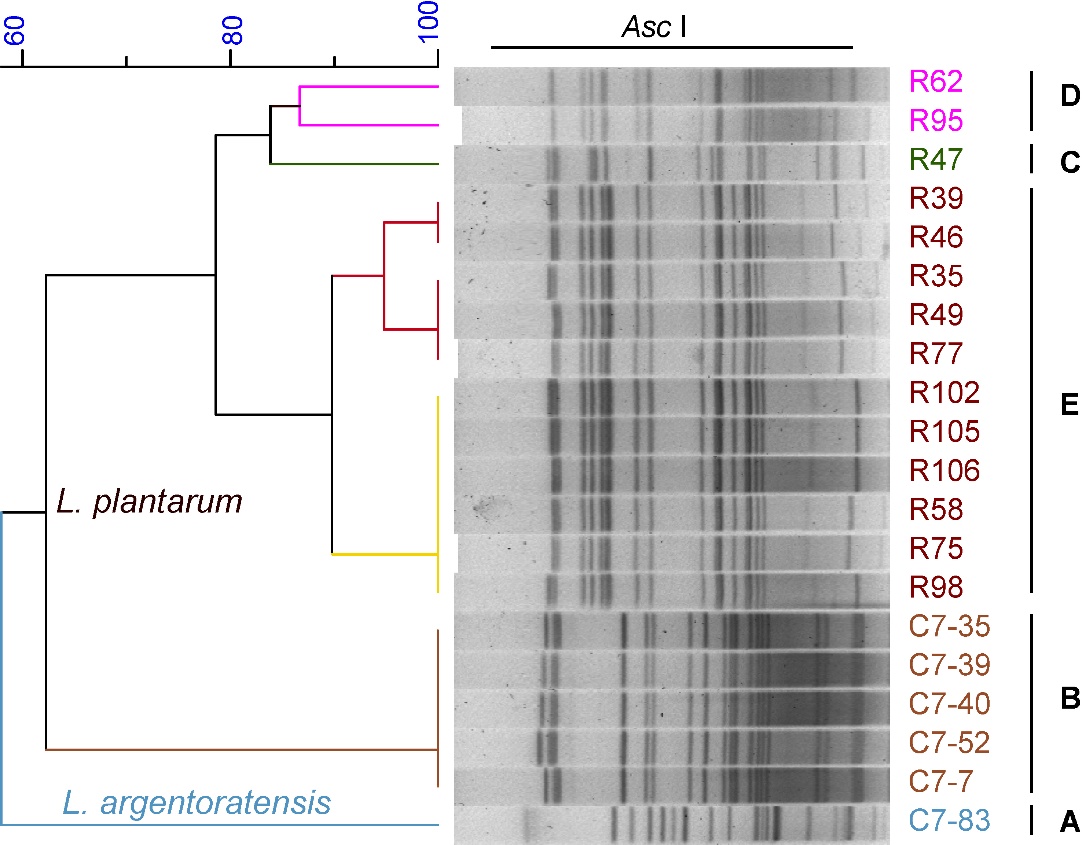


**Figure S2.** Dendrogram of the PFGE patterns of the 20 *Lactiplantibacillus* isolates. digested with restriction enzyme *Asc* I.


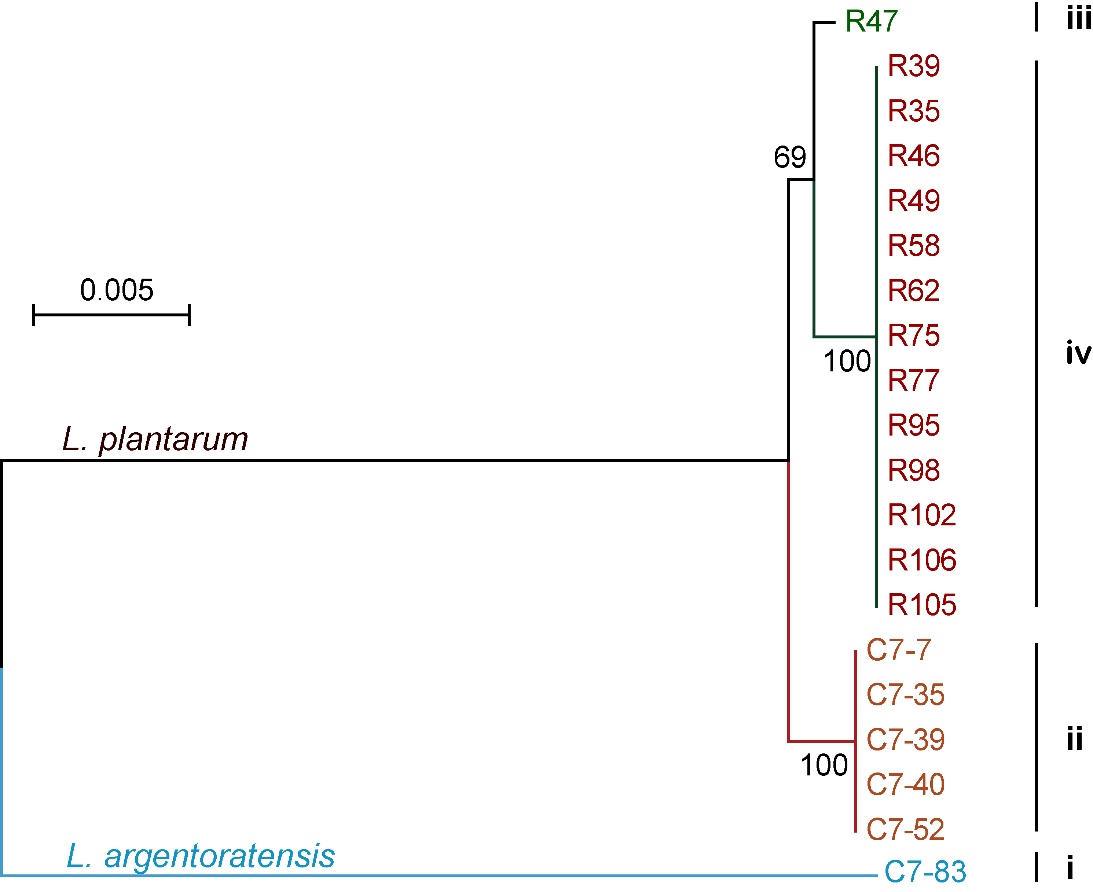


**Figure S3.** Phylogenetic tree of the 20 *Lactiplantibacillus* isolates constructed using WGS-based MLST.

**Figure S4.** Growth pattern of 10 representative *Lactiplantibacillus* isolates in MRS broth at 37 °C with pH = 7.4 or pH = 2.5 or in 0.3% bile acids for 18 h.
